# Life Cycle Assessment and Life Cycle Costing Analysis for Removing Per- and Polyfluoroalkyl Substances from Landfill Leachate with Foam Fractionation Technology

**DOI:** 10.1021/acsestwater.5c00381

**Published:** 2025-10-22

**Authors:** Gengyang Li, Yifei Wang, Qingguo Huang, Mason Peng, Ke Li

**Affiliations:** a College of Engineering, University of Georgia, Athens, Georgia 30602, United States; b College of Agricultural and Environmental Sciences, Griffin, Georgia 30223, United States

**Keywords:** life cycle assessment (LCA), life cycle costing analysis (LCCA), per- and polyfluoroalkyl substances (PFASs), foam fractionation (FF), landfill leachate (LL)

## Abstract

Understanding the environmental impacts and economic costs of treatment technologies is essential for developing sustainable strategies for managing per- and polyfluoroalkyl substances (PFASs). This study focuses on the treatment of PFAS-contaminated landfill leachate using foam fractionation (FF) technology. A parametrized life cycle assessment and life cycle costing analysis were conducted to evaluate the performance of one-stage and three-stage FF systems. Full-scale operational data and EPA design models were used to assess environmental and economic impacts based on a functional unit of treating 1000 m^3^ of PFAS-contaminated landfill leachate. The global warming potential was estimated at 818 kg CO_2_ eq for the one-stage system with 20% foam fraction, 357 kg CO_2_ eq for the one-stage system with 1% foam fraction, and 402 kg CO_2_ eq for the three-stage system with 1% foam fraction. Life cycle costs were estimated at $77.4 and $110.6 per functional unit for the one-stage and three-stage systems, respectively, using the net present value method. Sensitivity and scale-up analyses were also performed to evaluate the influence of operational parameters and system configurations on both environmental and economic outcomes.

## Graphical abstract

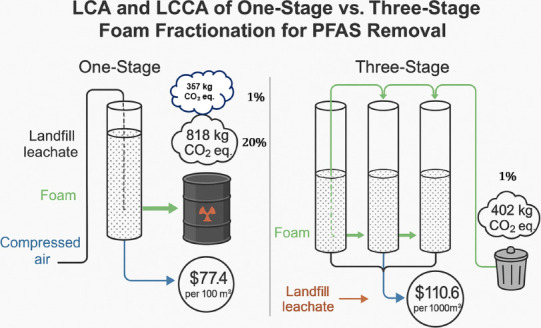

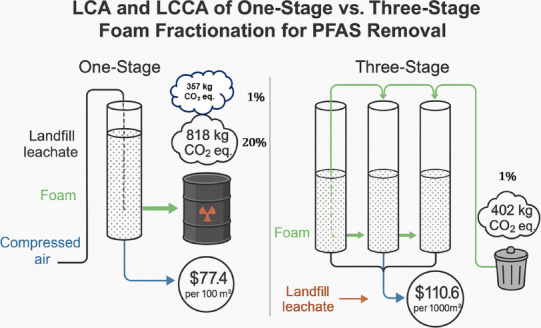

## 1 Introduction

Per- and polyfluoroalkyl substances (PFASs) have drawn public concern recently due to their toxic properties and environmental persistence.^1^ PFAS substances are a broad class of emerging environmental contaminants used heavily by the industrial, commercial, and manufacturing sectors in previous decades; some products include surface coating, lubricants, and firefighting foams.^2,3^ PFASs are highly persistent and bioaccumulated in the environment within biota and humans. Because of this, PFAS species have been consistently detected within the environment, in food products, and in humans.^4^ Various potential health effects have been attributed to PFASs exposure due to their high toxicity.^5^

Landfill leachate (LL) is a complex matrix containing high ammonia concentrations, chemical oxygen demand, salts, and chemicals. Discharged leachate water from landfills are an important contributor to the origin of PFASs in the environment, with total aqueous concentrations ranging from 100 to 100 000 ng/L.^6^ PFASs in landfills originate from discarded consumer and industrial waste or PFASs-contaminated biosolids.^7^ Moreover, landfilled bottom ash from waste incinerators may still contain incompletely combusted PFASs. Biological leaching and physicochemical desorption of these PFASs result in their release to the landfill leachate, leading to high aqueous PFASs concentrations. Although the production and use of some PFASs are banned or restricted, historically stored wastes in landfills are expected to last for a long time and remain a problem in the foreseeable future.

A variety of technologies have been applied for the removal of per- and polyfluoroalkyl substances (PFASs) from water, including granular activated carbon (GAC), ion exchange (IX), nanofiltration/reverse osmosis (NF/RO), and electrochemical oxidation (EO). GAC and IX are widely used for long-chain PFASs but exhibit limited effectiveness for short-chain compounds and require frequent media regeneration or replacement, leading to high life cycle costs and complex waste handling. Membrane-based processes like NF/RO offer broad PFAS rejection but are energy-intensive and generate large volumes of concentrate that still require disposal or destruction. EO, while promising as a destructive technology, typically demands high electrical energy input and is best suited as a polishing step for concentrated waste streams.

In comparison, foam fractionation (FF) is a promising separation-based technology that exploits the surface-active nature of PFASs, particularly long-chain species. Foam fractionation (FF) has been proven to effectively remove PFASs from PFASs-contaminated landfill leachate.^8^ Using fine air bubbles, foam fractionation concentrates PFASs from a water phase into a more minor volume foam phase. The foam phase contains highly concentrated PFASs that can be disposed of or further treated.^9^ The advantage of FF technology is that it can be implemented using a standard aeration process in wastewater treatment facilities. Converting an existing aeration process to foam fractionation for PFAS removal only requires installation of an appropriate foam collection system. PFASs removal depends on several operational parameters such as contact time, air ratio, air flow rate, and foam fraction.^10^ Removal efficiencies for long-chain PFASs are typically greater than 90% and removal of short-chain PFASs is typically 50% or less.^10^ FF as a potent remediation technology for landfill leachate has been reported at the lab-scale, pilot-scale, and commercial-scale (Table 1).^11,12^

**Table 1:** Literature Review of FF Operation Parameters Treating PFAS-Contaminated Landfill Leachate in Lab, Pilot, and Full Scales in the Recent Three Years (N Represents Not Reported)

references	flow mode	scale	vessel volume (L)	air flow rate (L/min)	air–water ratio	aeration time (min)
Wang et al.^13^	batch	lab	0.20	5	50	2
McCleaf et al.^15^	batch	lab	1.2 and 2.4	3.6, 7.1, and 15	15–180	5, 10, 20, 40, and 60
	continuous		2.4	15	31–124	5,10, and 20
Robey et al.^11^	batch	lab	1	2.6	N	N
Smith et al.^10^	continuous	pilot	46	5–20	2.2–13	10, 15, 20, and 30
Burns et al.^17^	semicontinuous	full	2600	N	N	21

A review of the FF operation parameters for lab, pilot, and full scales from literature in recent years is presented in Table 1. The air–water ratio, air flow rate, and aeration time are the major parameters that affect the PFASs treatment efficiency. All these studies found that PFASs of longer chain lengths were prone to higher removal and more significant enrichment than the short-chain. Also, the PFASs removal efficiency and the enrichment in the three-stage treatment were higher than in the one-stage FF treatment.^10,13–15^ Burns and his co-workers demonstrated a commercial-scale three-stage semibatch foam fractionation process, Surface-Active Foam Fractionation (SAFF), for the removal of PFASs from a landfill leachate catchment in Sweden.^16^ There have been 23 sampling events for PFASs influent and effluent concentrations over 10 months, during which approximately 80,000m^3^ of leachate feed has been successfully treated without the need for complex pretreatment.^17^ The contracted throughout was 330 m^3^ per day (120,000m^3^/year), but, in practice, it varied between 200 m^3^ and 500m^3^ per day depending upon the inventory of the upstream leachate catchment. Over the trial period, SAFF was successful in removing ≥ 98.7% PFOS (C8), ≥ 99.7% PFOA (C8), and ≥ 98.8% PFHxS­(C6) from the feed stream without using absorbent media or chemical amendment consumables. However, the removal efficiency was relatively low for short-chain PFASs.

Life cycle assessment (LCA) and life cycle costing analysis (LCCA) are methodologies for quantifying and comparing the systems-level environmental impacts and associated economic costs, respectively, of a product or process over a defined life cycle.^18^ Recent LCA studies have reported the toxicity of PFASs plumes, alternative surfactants to PFASs, membrane bioreactor treatment of PFASs in landfill leachate, and a hybrid ion exchange-electrochemical oxidation treatment trains.^19–21^ Foam fractionation (FF) has been applied in various contexts beyond PFAS remediation, including the treatment of surfactants, dyes, proteins, and other industrial effluents. However, life cycle assessment (LCA) and life cycle costing analysis (LCCA) studies of FF remain limited in scope. Most published LCAs on FF have focused on laboratory- or pilot-scale systems, with limited attention to full-scale system integration. Few publications have reported systems-level environmental or economic impacts associated with FF processes. For instance, Vo et al. (2023) reported energy demand and cost estimates for FF using cosurfactants, but their scope was restricted to operational energy inputs without consideration of capital construction, end-of-life waste handling, or economic trade-offs at scale.^12^ Similarly, Wang et al. (2023) investigated combined FF and electrochemical oxidation processes at the lab scale, but did not employ a parametrized model to assess system sensitivity to operational changes.^13^ However, a comprehensive LCA covering construction, operation, and end-of-life stages is still lacking in the published literature. Moreover, to our knowledge, no existing studies have evaluated FF’s environmental and economic trade-offs in comparison with other PFAS treatment technologies using a full-scale parametrized LCA framework. This study aims to address this gap by quantifying and comparing the life cycle environmental and cost impacts of one-stage and three-stage FF systems for PFAS removal from landfill leachate in full scale. Sensitivity analyses were then conducted to identify the system parameters and design decisions that are expected to have significant effects on the life cycle impact. Moreover, the scale-up analysis section discussed the life cycle impact on the functional unit at various system scales.

## 2 Materials & Methods

### 2.1 Goal and Scope Definition

Life cycle assessment (LCA) is an environmental impact assessment method that considers the material and energy interactions over the life cycle of a product or process that impacts the environment. The LCA methodology in this analysis follows international guidelines set by the International Standards Organization (ISO) in ISO 14040–14044. These guidelines set the framework for LCA and consist of four major components: goal and scope definition, life cycle inventory (LCI), life cycle impact assessment (LCIA), and interpretation of results.

This LCA and LCCA study aims to analyze the environmental impacts and economic costs of remediation of PFAS-containing landfill leachate with one-stage (20% and 1% foam fraction) and three-stage (1% foam fraction) FF systems. First, LCA models of one-stage and three-stage FF systems are built to assess the environmental impacts of treating PFAS-contaminated landfill leachate. The environmental impacts of the three scenarios are calculated and compared. Process contribution analysis is then performed to understand the key operational variables. Afterward, the economic costs for the two systems are calculated and compared in the life cycle of 30 years.

The design flow of 1 MGD (million gallons per day) for the FF treatment plant was selected based on values commonly reported in full-scale landfill leachate treatment systems, including field-scale foam fractionation deployments.^17^ This flow rate allows for a realistic full-scale basis while maintaining comparability across systems.

While the effluent quality after one-stage and three-stage FF may differ, particularly in short-chain PFAS concentrations, this study applies a functional unit of treating 1000 m^3^ of PFAS-contaminated landfill leachate and evaluates the life cycle burdens associated with the treatment process itself, up to the disposal of the foam waste. The impact of downstream effluent quality is excluded from the system boundary to maintain methodological focus on the comparative infrastructure and energy demands of the FF systems. This boundary is consistent with previous LCAs of partial-removal PFAS technologies and supports technology-level comparison.

There is only one vessel in the one-stage FF system (Figure 1). Compressed air flows from the bottom, and the high-concentration PFAS foam is generated during this aeration process. Then the foam (20% or 1% volume of the input LL) will be extracted by the vacuum from above and sent to the disposal unit, which is hazardous waste incineration. The 80% or 99% volume effluent comes out to be the clear treated LL. In the three-stage FF system, there are three vessels. The flow mode is designed to be a semibatch multistage, optimizing separation and concentration steps within a single system.^17^ After the FF treatment in the first stage, the foam extracted by the vacuum from above (around 20% volume of the input LL) goes to the vessel in the second stage (green line in Figure 2).^17^ Then, the foam (0.0154% volume of the input LL) produced in the second stage goes to the third stage for further treatment.^17^ At the same time, the LL bottom solutions of second stage and the third stage goes back to the vessel in the first stage (orange lines in Figure 2). Finally, a highly concentrated PFASs aqueous liquid waste forms and will be treated as hazardous waste by incineration, whose volume is around 0.00006% volume of the initial input LL.^17^

**Figure 1:**
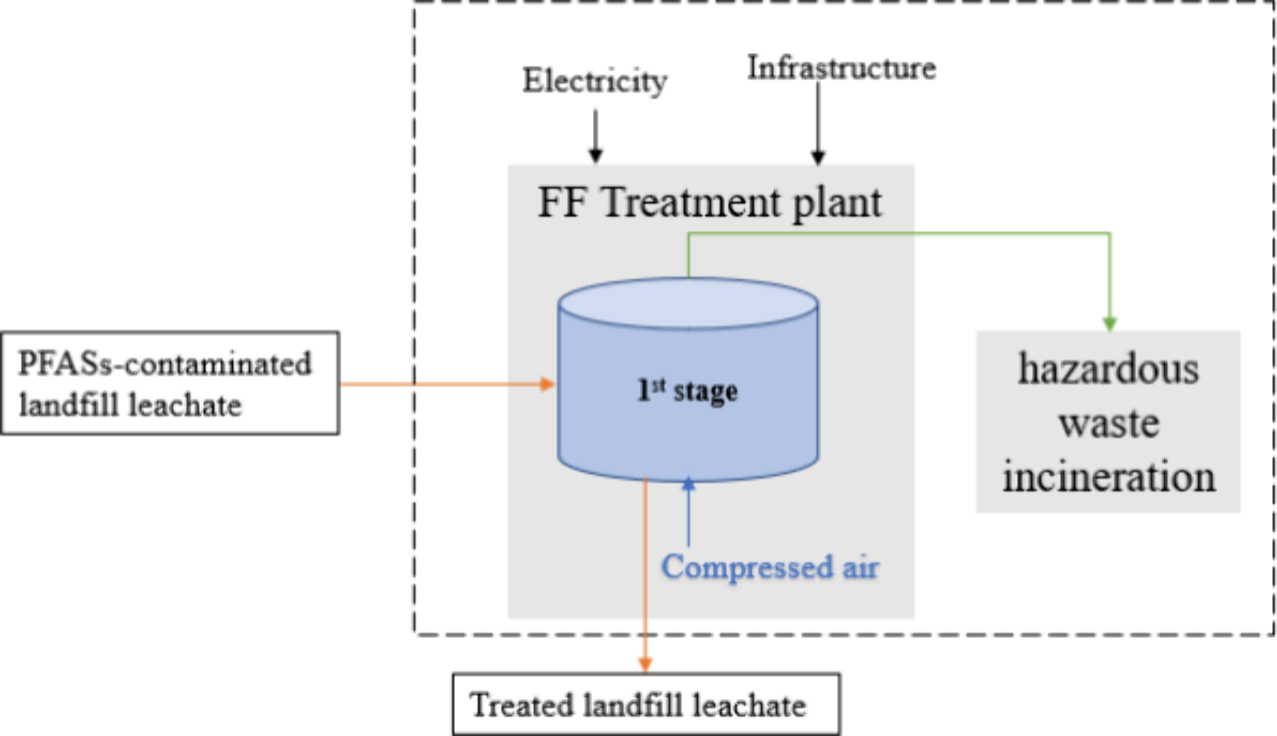
System boundary of the one-stage FF system (20% and 1%) for PFASs treatment; the orange line is the landfill leachate flow; the green line is the foam flow; the blue line is the compressed air flow.

**Figure 2:**
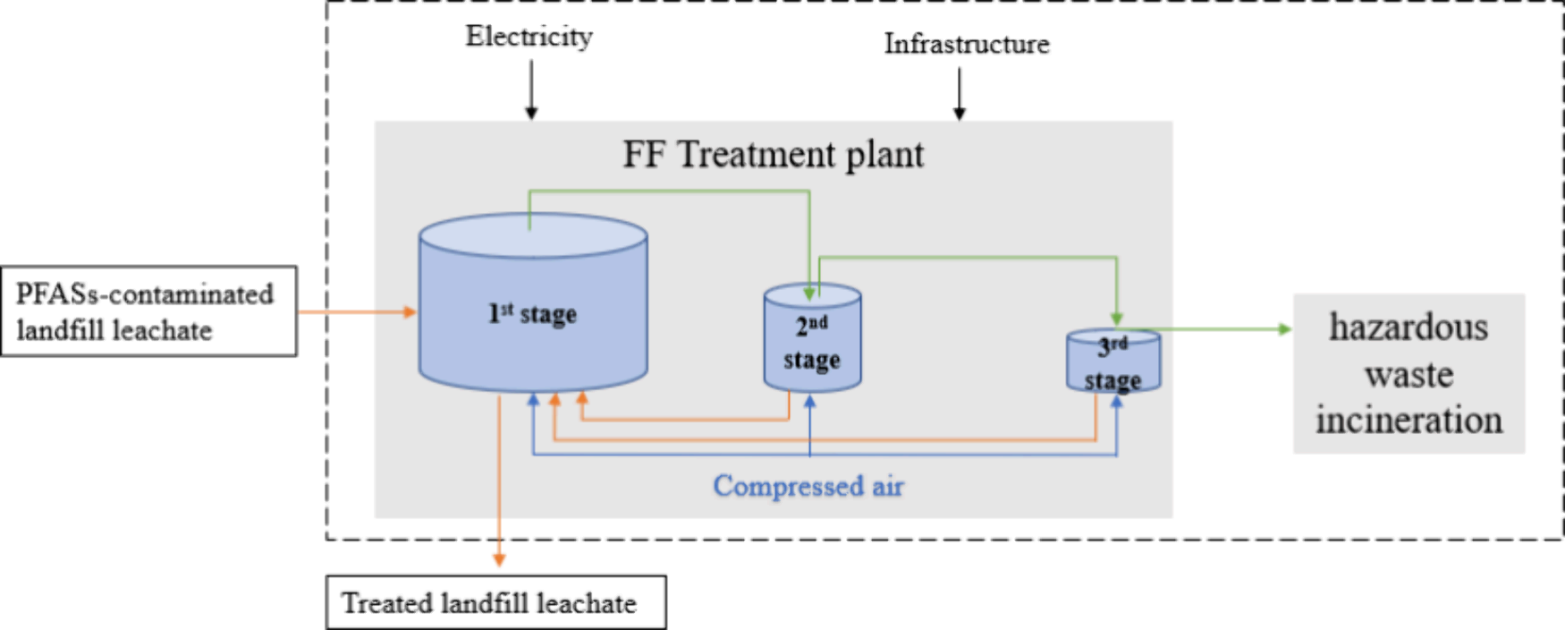
System boundary of the three-stage FF system for PFASs treatment; the orange line is the landfill leachate flow; the green line is the foam flow; the blue line is the compressed air flow.

The functional unit chosen for the analysis is treating 1000 m^3^ PFASs-contaminated landfill leachate. The design flow of the FF treatment plant is determined to be 1 MGD (million gallons per day). The overall system boundaries are shown in Figure 1. The diagram includes the one-stage and three-stage FF treatment systems.

### 2.2 Life Cycle Assessment Methodology

In this study, SimaPro 7.0 software was used to build the LCA model with the Eco-invent and USA INPUT OUTPUT 2002 database to perform the environmental impact assessment using EPA’s Tool for Reduction and Assessment of Chemicals and Other Environmental Impacts (TRACI) methodology. Foreground inventory data for foam fractionation system performance—including compressed air requirements, vacuum usage, and foam waste volumes—were obtained from Burns’s commercial-scale study, energy audit data from municipal treatment plants, and EPA design models. Capital and maintenance cost estimates were derived using the WBS model for multistage bubble aeration systems, supplemented by vendor data and regional engineering reports.^22,24,26^

This study employed a parametrized LCA framework, in which key inventory inputs were defined as functions of operational parameters to reflect real-world variability in system design and performance. Specifically, inputs such as foam fraction, compressed air use (based on AWR), electricity demand, and hazardous waste output were modeled using equations derived from full-scale study. For example, compressed air input was calculated based on the air–water ratio and system flow rate, while hazardous waste volume was linked to foam fraction and stage configuration. This parametrization allowed for scenario analysis (e.g., 1% vs 20% foam fraction) and scale-up modeling across treatment capacities ranging from 0.1 to 10 MGD. The resulting parametrized LCA model enabled us to evaluate how different operational strategies affect environmental impacts per 1000 m^3^ of treated landfill leachate.

LCIA is a qualitative and quantitative assessment of the environmental impact of a process based on the resource, energy consumption data, and various emission data. A total of 7 environmental impact categories were considered in this study, including ozone depletion (OD, CFC-11 equiv), global warming potential (GWP, kg CO_2_ eq), acidification (AD, mol H^+^ eq), marine eutrophication (ME, kg N eq), smog formation (SF, kg O_3_ eq) and ecotoxicity (ET, CTUe), carcinogens (C, CTUh), noncarcinogens (NC, CTUh), and respiratory effects (RE, kg PM_10_ eq) (Table S1).

### 2.3 Life Cycle Cost Analysis (LCCA) Methodology

The life cycle inventory for the FF remediation system was also used to estimate costs for treatment, both in actual dollars and on a per-unit-volume basis ($ per 1000 m^3^ treated). Unit costs include capital construction costs, operation costs (electricity used for vacuuming foam and generating compressed air), and maintenance costs. The costs for capital construction and maintenance (labor) were estimated using the EPA-derived work breakdown structure (WBS) for multistage bubble aeration treatment.^22^ The operation cost (electricity) was estimated based on a previous energy report for a wastewater treatment plant.^23^

In a 30-year life cycle, the inflation-adjusted initial costs, maintenance and rehabilitation costs, and salvage value being used can be indicated with the Net Present Value (NPV).^18^ The NPV (eq 1) can be applied to the FF treatment system case,

$$\mathrm{NPV}=\mathrm{Initial}\;\mathrm{Construction}\;\mathrm{cost}+\sum_{K=1}^{N}(\mathrm{Operation}\;\mathrm{cost}+\mathrm{Maintenance}\;\mathrm{cost})\left[\frac{1}{(1+i{)}^{k}}\right]-\mathrm{Salvage}\;\mathrm{Value}\left[\frac{1}{(1+i{)}^{N}}\right]$$ 1

where: Initial construction cost and maintenance cost derive from the EPA WBS model; operation cost is calculated based on the electricity used for compressed air and vacuum; N = analysis period in years (N = 30 in this study); i = discount rate in percent (i = 9.8% in this study); k = number of years from the initial construction to the K^th^ expenditure;

### 2.4 Inventory Data collection

The LCA inventory data collection includes capital construction, vacuum electricity, compressed air generation, and hazardous waste disposal (Table 2). The LCCA inventory data collection includes all of the LCA inventory items and maintenance costs.

**Table 2:** Inventory Data and Environmental Database Used for the One-Stage FF (20 and 1%) System and Three-Stage FF System^a^

inventory item	one-stage FF (20%)	one-stage FF (1%)	three-stage FF	units	item name in SimaPro	database
capital construction	22.12*	22.12*	29.30*	$	other nonresidential structures	USA INPUT OUTPUT 2002
electricity for vacuum	15.72	15.72	18.87	kWh	electricity, medium voltage, at grid, US/U	Eco-invent
compressed air	4300	4300	5161	m^3^	compressed air, optimized generation, > 30 kW, 6 bar gauge, at compressor/RER U	Eco-invent
hazardous waste disposal	200	10	0.0006	m^3^	disposal, hazardous waste, 25% water, to hazardous waste incineration/kg/CH	Eco-invent

a *The capital construction cost was converted to the currency value in 2002 to perform LCA analysis with SimaPro software.

The capital construction cost used in the inventory is built from the EPA-derived Work Breakdown Structure (WBS) for multistage bubble aeration treatment, seeing details in the Appendix section 1 for one-stage and section 2 for three-stage.^22^ Also, as for the scale-up analysis, the total construction cost is determined by Eq. 2).^23^ Costs are correlated in terms of a base cost multiplied by a ratio of sizes raised to the power n. The Q is flow rate or capacity. In this study, n is determined to be 0.8 for foam fractionation (Woods, 2007).

$${\mathrm{cost}}_{\mathrm{scales}}={\mathrm{cost}}_{\mathrm{ref}}\times {\left(\frac{Q_{\mathrm{scaled}}}{Q_{\mathrm{ref}}}\right)}^{n}$$ 2

The operation parameters are referenced in Smith et al.’s pilot study and Burns et al.’s full-scale field study.^17^ The operational input for FF is mainly from blowing the compressed air, which can be calculated using the Air–Water Ratio (AWR). The AWR was assumed to be 4.3, according to Smith’s pilot study.^10^ The foam flow in the three-stage FF system was determined by the commercial filed FF study of Burns et al., which as described in Section 2.1.^10^ The electricity input was estimated based on a previous municipal wastewater treatment plant energy evaluation report for Tonawanda town.^23^ According to the EPA-WBS model, the average electricity price for water treatment was estimated to be $0.11.^22^ The maintenance cost was estimated using the EPA-derived work breakdown structure (WBS) for multistage bubble aeration treatment.^22^

## 3 Results and Discussion

This section analyzes the environmental impact and economic cost assessment results of the one-stage and three-stage FF treatment systems following the methodology described in Section 2. All impact assessment results are presented per functional unit, treating 1000 m^3^ PFASs-contaminated landfill leachate.

### 3.1 Environmental Impact Assessment Analysis

#### 3.1.1 Environmental Impact Assessment Results and Comparison

The environmental impacts of the foam fractionation (FF) systems—one-stage with 20% foam fraction, one-stage with 1% foam fraction, and three-stage configuration—were assessed using the life cycle assessment (LCA) methodology. Table 3 summarizes the total environmental impacts across multiple categories for each system per functional unit of 1000 m^3^ treated landfill leachate. Table S2, S3, and S4 also show the breakdown impact values from the inventory items for the one-stage FF system (20% and 1%) and the three-stage FF system, respectively.

**Table 3:** Total Environmental Impact Results for One-Stage (20% and 1%) and Three-Stage FF Systems with the Functional Unit

impact category	one-stage FF system (20%)	one-stage FF system (1%)	three-stage FF system	unit
ozone depletion (OD)	5.60 × 10^–5^	3.00 × 10^–5^	3.56 × 10^–5^	kg CFC-11 eq
global warming potential (GWP)	8.18 × 10^2^	3.57 × 10^2^	4.02 × 10^2^	kg CO_2_ eq
smog formation (SF)	2.15 × 10^1^	1.39 × 10^1^	1.62 × 10^1^	kg O_3_ eq
acidification (AD)	1.11 × 10^2^	8.14 × 10^1^	9.60 × 10^1^	mol H^+^ eq
marine eutrophication (ME)	3.84 × 10^0^	2.54 × 10^0^	2.97 × 10^0^	kg N eq
non-carcinogenics (NC)	1.09 × 10^–4^	3.71 × 10^–5^	4.01 × 10^–5^	CTUh
respiratory effects (RE)	1.24 × 10^–4^	8.22 × 10^–5^	9.66 × 10^–5^	CTUh
ecotoxicity (ET)	4.89 × 10^–1^	3.70 × 10^–1^	4.37 × 10^–1^	kg PM_10_ eq
ozone depletion (OD)	1.38 × 10^3^	6.19 × 10^2^	7.00 × 10^2^	CTUe

The original one-stage FF system (20% foam fraction) exhibits the highest environmental burden across most categories. Notably, its global warming potential (GWP) is 818 kg CO_2_ eq., and ecotoxicity is 1380 CTUe—both considerably higher than those of the other scenarios. This is largely attributed to the large volume (200 m^3^) of hazardous foam waste requiring incineration.

In contrast, the three-stage FF system shows consistently lower environmental impacts, with a GWP of 402 kg CO_2_ eq. and ecotoxicity of 700 CTUe. These reductions result primarily from the substantial volume reduction of hazardous waste (to 0.0006 m^3^), along with energy efficiency gains from process design.

Importantly, the one-stage FF system with a reduced foam fraction of 1% demonstrates significantly improved environmental performance compared to the 20% case. Its GWP drops to 357 kg CO_2_ eq., which is lower than that of the three-stage system, and the ecotoxicity impact is also reduced to 619 CTUe. These findings suggest that operational optimization—particularly minimizing foamate volume—can substantially reduce environmental burdens, making the one-stage configuration a competitive alternative to multistage systems in certain contexts.

Across all impact categories, the 1% foam fraction scenario consistently shows lower or comparable values to the three-stage system. This underscores the critical role of foam fraction volume in driving the environmental impacts of FF processes and highlights the potential benefits of optimizing foam separation performance, even in simpler system architectures.

Overall, while the three-stage FF system generally performs better than the conventional one-stage design with 20% foam fraction, the one-stage system with 1% foam fraction achieves equal or superior environmental outcomes, emphasizing that both system configuration and operational parameters must be considered together in technology selection.

#### 3.1.2 Contributions to Impacts

Figure 3 shows the relative contributions to the environmental impact of each inventory item, including capital construction, electricity for vacuum, compressed air, and hazardous waste disposal. Hazardous waste disposal and compressed air contribute significantly to the one-stage FF system. The hazardous waste also contributes the most regarding ozone depletion, global warming potential, carcinogens, and ecotoxicity impact. The compressed air contributes most to smog formation, acidification, marine eutrophication, noncarcinogens, and respiratory. However, the disposal has little environmental impact on three-stage FF system because there is little hazardous waste foam at the end. In contrast, compressed air contributes most in all impact categories (>90% except for the ozone depletion category).

**Figure 3:**
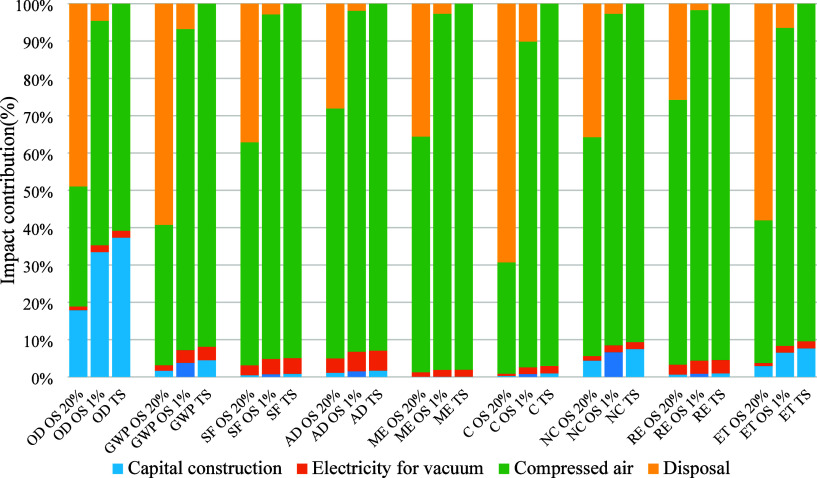
Relative contributions of each inventory item to the total environmental impacts in the one-stage (OS) FF (20 and 1%) and three-stage (TS) FF treatment system (note: OD, ozone depletion; GWP, global warming potential; SF, smog formation; AD, acidification; ME, marine eutrophication; C, carcinogens; NC, noncarcinogens; RE, respiratory; ET, ecotoxicity).

#### 3.1.3 Impact Normalization and Interpretation

Unit normalization is employed to facilitate direct comparison among environmental impact categories of varying magnitudes. This standardization utilizes ‘impact per person per year’ data aggregated for the average American citizen in a given year (2008); the analysis uses this to divide the raw impact quantity in each category thereby yielding units presented in “person-years”. This method enables comparison across all categories.

Table 4 presents impact data of the three scenarios for all impact categories normalized in terms of these person-year units.^25^ Presenting the data in this way reveals that acidification is the most impactful category relative to the average US emissions. Among all categories, acidification exhibits the highest relative impact when normalized against average U.S. emissions. This suggests that acidification may serve as a critical indicator for identifying and optimizing environmentally sensitive parameters in system design and operation. This elevated impact is primarily attributed to the intensive energy consumption during compressed air generation, which releases nitrogen oxides (NO_*x*_) and sulfur dioxide (SO_2_) as byproducts of electricity production. These acidic precursors are known contributors to environmental acidification. Since foam fractionation relies heavily on aeration using compressed air across multiple stages or high flow rates, the indirect emissions from grid electricity used to power air compressors become a dominant driver in this category, particularly when the regional energy mix includes fossil fuel-based sources like coal and natural gas. Furthermore, the normalized comparison shown in Figure S1 highlights that the three-stage FF system consistently results in lower environmental impacts across all categories compared to the one-stage system with 20% foam fraction. These results further support the environmental advantages of the three-stage configuration, particularly when considering long-term sustainability goals.

**Table 4:** Environmental Impact Normalized Values for the One-Stage (20 and 1%) and Three-Stage FF Treatment Systems (Person-Years per 1000 m^3^ Treated)

impact category	one stage (20%)	one stage (1%)	three stage	unit
ozone depletion (OD)	0.00035	0.00019	0.00022	person-years
global warming potential (GWP)	0.034	0.015	0.017	person-years
smog formation (SF)	0.015	0.010	0.012	person-years
acidification (AD)	12.20	0.89	10.54	person-years
marine eutrophication (ME)	1.74	0.12	1.35	person-years
non-carcinogenics (NC)	2.15	0.74	0.79	person-years
respiratory effects (RE)	0.12	0.079	0.093	person-years
ecotoxicity (ET)	0.20	0.015	0.18	person-years
ozone depletion (OD)	0.13	0.056	0.064	person-years

#### 3.1.4 Sensitivity Analysis for Environmental Impact

The life cycle assessment presented above compared the environmental impacts of two full-scale foam fractionation (FF) systems. However, operational parameters, particularly those related to energy inputs, can significantly influence environmental performance. To identify the most impactful design and operational factors, a sensitivity analysis was conducted.

Among all inputs, compressed air generation emerged as a key contributor to environmental impacts in both the one-stage and three-stage FF systems. Due to its central role in the aeration process, variations in the power requirements and operating pressure of air compressors can substantially affect emissions across multiple impact categories.

To explore this sensitivity, we analyzed the unit environmental impacts of compressed air generation under various power and pressure conditions, referencing data for systems operating at >30 kW and 6 bar gauge as the baseline

Table S4 provides the unit environmental impact of compressed air generation under five different operating conditions. Figure S2 illustrates the normalized environmental impact of 1m^3^ compressed air under different generation conditions. The results show that higher pressure and lower power efficiency (<30 kW) dramatically increase environmental burdens across all categories. For instance, GWP values increase from 0.0715 kg CO_2_ eq/m^3^ at >30 kW, 6 bar gauge to 0.199 kg CO_2_ eq/m^3^ at <30 kW, 12 bar gauge—an increase of nearly 180%. Similarly, acidification (AF) impact triples, and ecotoxicity (ET) increases by a factor of 10. Although it may initially appear that using a lower power compressor would reduce energy consumption and emissions, Table S4 reveals the opposite: environmental impacts increase significantly under lower power conditions. This is because smaller compressors (<30 kW) are generally less energy-efficient, requiring more electricity to generate the same volume of compressed air. The higher energy demand arises from greater relative friction losses, suboptimal motor design, and reduced thermal recovery capabilities. As a result, key impact categories such as global warming potential, acidification, and ecotoxicity show marked increases under low-power, high-pressure configurations—highlighting the importance of energy efficiency in compressed air system design.

These results explain the high sensitivity of the foam fractionation system to air generation conditions and underscore the importance of optimizing compressor performance for reducing system-wide environmental burdens. Overall, the results emphasize that minimizing energy intensity in compressed air production is critical for reducing the environmental footprint of FF-based PFAS treatment systems.

#### 3.1.5 Effect of Foam Fraction on Treatment Performance

While the life cycle analysis in this study focuses on environmental and economic metrics, changes in foam fraction also influence PFAS removal efficiency. Literature reports (Table 1) indicate that higher foam fractions generally result in greater capture of short-chain PFASs, which are less surface-active and require larger processed volumes for efficient separation.^10,17^ For example, pilot-scale continuous FF achieved >90% removal of long-chain PFASs (≥C8) at both 1% and 20% foam fractions, but short-chain PFAS (≤C6) removal dropped from ∼ 60% at 20% foam fraction to ∼ 40% at 1% foam fraction.10 In three-stage configurations, sequential concentration partially compensates for the lower short-chain removal in each stage, yielding higher overall performance. These findings suggest that while reducing foam fraction from 20% to 1% can substantially improve environmental performance by lowering hazardous waste volume and compressed air demand, it may also reduce treatment efficacy for certain PFAS species—particularly short-chain compounds—unless additional polishing steps are employed. This trade-off should be considered in technology selection, especially where regulatory discharge limits apply to a broad PFAS spectrum.

### 3.2 Life Cycle Costing Assessment Analysis

#### 3.2.1 Life Cycle Costing Assessment Results and Comparison

The LCCA results for the one-stage (20% and 1%) and three-stage FF systems are in Table S6. The unit costs were calculated using NPV equation in a 30-year life cycle according to the methodology described in section 2.3. The economic cost assessment included capital construction cost, operation cost (electricity), and maintenance cost (labor).

The comparison of the life cycle costing between the one-stage FF system (20% and 1%) and the three-stage FF treatment system is shown in Figure 4. The maintenance cost for the three-stage FF system is 66% more than the one-stage FF system with the functional unit. However, the operation cost for the three-stage FF system has a smaller difference, which cost 25% more than the one-stage FF system. The capital construction cost for the three-stage FF system is around 32% more than the one-stage FF system with the functional unit. These results indicate that while the three-stage system offers notable environmental benefits, it incurs a higher total life cycle cost. This trade-off should be considered when evaluating full-scale implementation scenarios.

**Figure 4:**
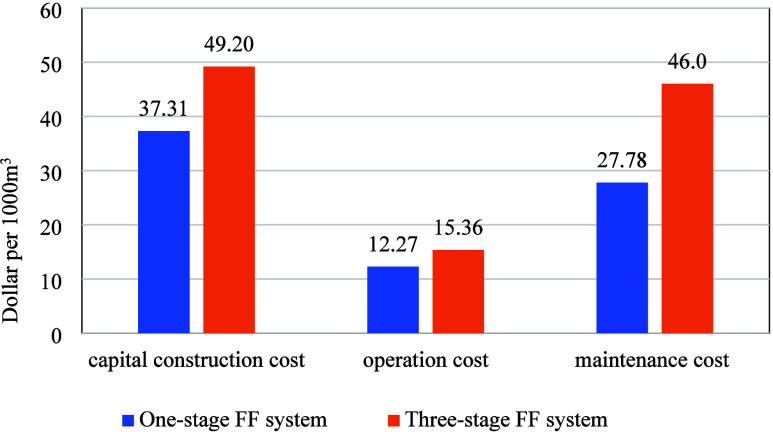
Life cycle costing comparison of the one-stage FF system (20 and 1%) and three-stage FF treatment system with the functional unit.

#### 3.2.2 Sensitivity Analysis for Economic Cost

In addition to environmental impacts, variations in system design can significantly affect the overall economic cost of foam fractionation (FF) treatment systems. To evaluate these influences, a sensitivity analysis was conducted focusing on capital construction costs, which represent a substantial portion of the life cycle cost.

The capital cost is determined by several design choices, including the number and size of vessels, pumps, blowers, piping, and control systems. To assess how different design references affect cost estimation, we compared values from four sources for treating 1000 m^3^ of PFAS-contaminated landfill leachate using a one-stage FF system: EPA WBS model (2022): $37.31, Woods (2007): $18.31, Westech (2016): $21.90, and Minnesota Pollution Control Agency (2023): $161.6. These results, summarized in Table S6, highlight a variation of nearly 50% depending on the design reference used. Such a spread demonstrates the sensitivity of capital cost estimates to assumed equipment specifications and data sources.

The analysis suggests that capital cost is a major driver of total system cost, and careful selection of design assumptions is essential for accurate life cycle costing. For real-world applications, site-specific design data should be incorporated whenever possible to improve cost accuracy.^26^

### 3.3 Scale-up Analysis

To evaluate the scalability of foam fractionation (FF) systems for PFAS remediation, we analyzed how the system size affects the global warming potential (GWP) per functional unit (1000 m^3^ treated). This section presents scale-up results for both one-stage and three-stage FF systems, based on operational and economic data from full-scale case studies.

According to the analysis above in section 3.1, the overall environmental impact is largely due to the capital construction cost, vacuum and compressed air electricity, and hazardous waste disposal. The capital construction impact was scaled using the power law equation for cost estimation in Equation 2) in section 2.4. The GWP contribution from capital construction is then:

$${\mathrm{GWP}}_{\mathrm{captial}}={\mathrm{cost}}_{\mathrm{scaled}}\times 0.61(\mathrm{kg}\;{\mathrm{CO}}_{2}\;\mathrm{eq}/\$)$$ 3

The compressed air GWP is modeled using the air–water ratio (AWR), flow rate, and an emission factor of 0.714 kg CO_2_ eq/kWh for electricity (based on the Southeastern U.S. grid mix). Incineration-related GWP is modeled using a foam volume reduction factor for each FF stage and an emission factor of 2.42 kg CO_2_ eq/m^3^ (Eco-invent database, hazardous waste incineration). The total GWP for each system includes vacuum energy, compressed air, and incineration for one-stage and three-stage FF system as follows:

$${\mathrm{GWP}}_{1‐\mathrm{stage}}=0.61\times C_{1}\times S^{n}+12.12+(0.715\times \mathrm{AWR}\times 1000)+\left(2.42\times \frac{1000}{x}\right)$$ 4

$${\mathrm{GWP}}_{3\mathrm{‐stage}}=0.61\times C_{3}\times S^{n}+14.55+(0.715\times \mathrm{AWR}\times 1000)\times \left(1+\frac{1}{x}+\frac{1}{y}\right)+\left(2.42\times \frac{1000}{z}\right)$$ 5

Where: C_1_ = 22.12 and C_3_ = 29.30 (capital costs at baseline); S is the scale factor (e.g., 2 for 2 MGD); AWR = air–water ratio (unitless); *n* = 0.8, the cost-capacity scaling exponent for FF; 0.715 kg CO_2_ eq/m^3^ is the emission factor for compressed air; 2.42 kg CO_2_ eq/m^3^ is the emission factor for incineration; x, y, z are enrichment factors for foam volume reduction per stage.

### 3.4 Disposal Methods Alternative

In addition to incineration, electrochemical oxidation (EO) has recently emerged as a promising method for destroying PFASs in the concentrated foam solution generated by foam fractionation.^27^ When EO is used following the FF process, the global warming potential (GWP) per 1000 m^3^ of treated landfill leachate can be estimated for both the one-stage and three-stage systems as a function of system scale and air–water ratio (AWR). The equations below estimate GWP (in kg CO_2_ eq ) for each system:

$$\mathrm{GWP}3=0.61\times C_{1}\times S^{n}+12.12+0.715\times \mathrm{AWR}\times 1000+\mathrm{EE}/O\times \mathrm{OM}\times \left(\frac{1000}{x}\right)\times 0.714$$ 6

$$\mathrm{GWP}4=0.61\times C_{3}\times S^{n}+14.55+0.715\times \mathrm{AWR}\times 1000\times \left(1+\frac{1}{x}+\frac{1}{y}\right)+\mathrm{EE}/O\times \mathrm{OM}\times \left(\frac{1000}{Z}\right)\times 0.714$$ 7

EE/O (kWh/m^3^) is the electric energy required to degrade PFASs by 1 order of magnitude in a unit volume; *OM* is the order of removal; 0.714 (kg CO_2_ eq/kWh) is the GWP of unit electricity (1 kWh) based on the energy mix of southeast U.S.

For both FF systems, the primary difference between GWP calculations lies in the disposal method. To assess the conditions under which EO becomes more sustainable than incineration, we analyzed the threshold at which the EO-related GWP exceeds that of incineration. It was determined that Incineration is more favorable when EE/O > 3.39 kWh/m^3^ for one log-order removal. Since many EO processes currently exceed this energy intensity, incineration remains the lower-GWP option in most practical scenarios. However, advances in EO energy efficiency or renewable electricity source could shift this balance in the future.

Although this study initially compared incineration and electrochemical oxidation (EO) based on their respective energy demands in kWh/m^3^, it is important to note that such a comparison is only valid when the energy sources are similar in carbon intensity. Incineration typically uses fossil fuels (e.g., natural gas, diesel), which have a higher GWP per unit energy than electricity in most regional grids. In contrast, EO relies on electricity, and its environmental performance varies significantly depending on the electricity mix. For example, as shown in Figure 7, electricity from the southeastern U.S. grid (used in this study) results in a GWP of 0.714 kg CO_2_ eq/kWh, while in countries with greener mixes, such as Sweden or France, the GWP per kWh is significantly lower. Under these conditions, EO may become the more environmentally favorable option—even with a higher energy intensity (EE/O). Therefore, regional energy characteristics must be considered when comparing disposal technologies for PFAS-contaminated foam.

### 3.5 Impact of Different Energy Mixes

As we see in eqs 3) to ([Disp-formula eq7]) and many life cycle analysis literature, the choice of energy source may significantly change the impact results for PFAS degradation. The 0.714 kg CO_2_ eq/kWh is GWP of 1 kWh of electricity from the energy mix of the southeast U.S., which consists of 37% natural gas, 34% coal, 24% nuclear, and 7% renewable energy, including hydro, wind, biomass and solar energy (DOE, 2015). As indicated in Figure 5, this number is relatively high compared to the energy mix worldwide. Therefore, if the energy mix used to supply the power for the EO process has more renewable resources, the overall GWP of the above scenarios will shift in favor of/EO. To apply the equations above to cases with different energy mixes, appropriate values for the GWP for each kWh energy consumption need to be used to replace the 0.714 kg CO_2_ eq/kWh.

**Figure 5:**
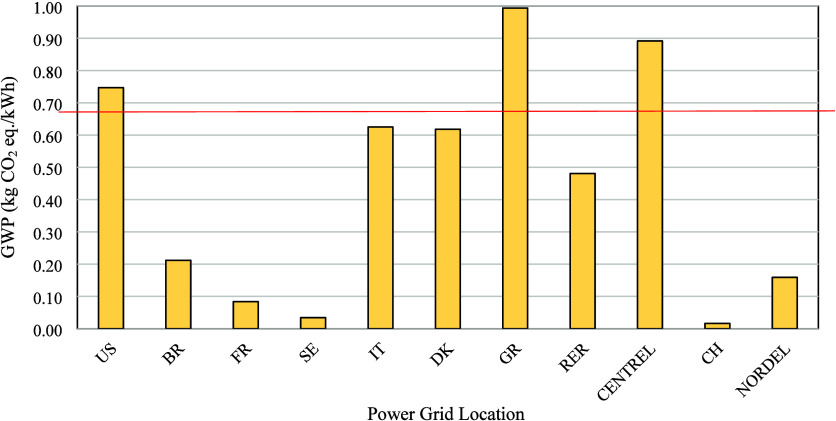
Variation in GWP values from different energy mixes. Abbreviations include: United States (US), Brazil (BR), France (FR), Sweden (SE), Italy (IT), Denmark (DK), Greece (GR), Average European (RER), Average Central American (CENTREL), Switzerland (CH), and Nordic Countries, excluding Iceland (NORDEL). Red line represents the number used in the equations that uses the southeast energy mix.

### 3.6 Comparative Assessment with Other PFAS Treatment Technologies

To contextualize the sustainability performance of foam fractionation (FF), we compared its life cycle environmental impacts and costs with those reported in the literature for other PFAS remediation technologies, particularly granular activated carbon (GAC) and ion exchange (IX) systems and their combinations with electrochemical oxidation (EO).

Ellis et al. (2023)^28^ conducted a comprehensive LCA and LCCA of GAC and IX systems for PFAS-contaminated groundwater. Their findings indicate that the GWP of GAC and IX systems ranges from 30 to 441 kg CO_2_ eq per 1000 m^3^, depending on whether the system used regeneration or media disposal. However, the life cycle costs were higher, ranging from $280 to $500 per 1000 m^3^ treated. In contrast, our study found that the three-stage FF system resulted in 402 kg CO_2_ eq per 1000 m^3^, and the optimized one-stage FF system with 1% foam fraction achieved 357 kg CO_2_ eq , with lower costs of $110.6 and $77.4, respectively. While the upper-end environmental impacts of IX and GAC are higher, their lower-end values can outperform FF in GWP—but not in cost.

Li et al. (2022) evaluated a hybrid IX-EO treatment train and reported notably lower environmental impacts, with GWP ranging from 72 to 84 kg CO_2_ eq per 1000 m^3^ treated, depending on the electricity mix and PFAS loading. This is substantially lower than the values reported for both FF systems in our study. The lower GWP in their study is largely attributed to optimized EO energy efficiency and the use of lower-impact electricity sources. However, their study also emphasized high operational energy demands and brine management costs, which can limit practical scalability and cost-effectiveness.

Additionally, Lin et al. (2025) examined the environmental trade-offs of thermal oxidation and pyrolysis of PFOS-loaded GAC.^29^ They found that thermal oxidation of spent GAC leads to GWP values varying from 5.8 ∼ 14 kg CO_2_ eq , primarily due to fossil fuel combustion during high-temperature treatment. In contrast, foam fractionation generates a much smaller waste volume, especially in the three-stage configuration (<0.0006 m^3^ per 1000 m^3^ treated), and enables modular disposal options, including electrochemical oxidation under green electricity conditions.

Overall, these comparisons emphasize that FF—especially when operated in multistage mode or optimized for low foam fractions—offers superior environmental performance and cost-efficiency relative to conventional GAC or IX systems. However, it is important to note that FF is more effective for long-chain PFAS, while IX offers broader removal capabilities, particularly for short-chain compounds. A hybrid treatment train may thus be necessary in practice.

## 4 Conclusions

A sustainable approach to managing PFAS-contaminated landfill leachate requires understanding the trade-offs between environmental impact and economic cost across different treatment technologies. This study conducted a life cycle assessment (LCA) and life cycle cost analysis (LCCA) of foam fractionation (FF) treatment systems using three configurations: a one-stage FF system with 20% foam fraction, a one-stage FF system with 1% foam fraction, and a three-stage FF system.

The conventional one-stage FF system with 20% foam fraction exhibited the highest environmental impacts across all categories, primarily due to the large volume of hazardous foam waste (200 m^3^ per 1000 m^3^ treated). In contrast, the three-stage FF system significantly reduced environmental burdens—cutting the global warming potential (GWP) by more than 50% and hazardous waste generation by several orders of magnitude (to 0.0006 m^3^). However, this environmental benefit came at a higher total cost, with capital, operation, and maintenance expenses exceeding those of the one-stage system by 32%, 25%, and 66%, respectively.

Importantly, reducing the foam fraction to 1% in the one-stage FF system led to major improvements in environmental performance. The GWP dropped to 357 kg CO_2_ eq , and the ecotoxicity impact was reduced to 619 CTUe—both values lower than and comparable to the three-stage system. These results highlight that operational optimization, particularly minimizing foam volume, can make single-stage systems environmentally competitive with multistage designs while maintaining lower overall cost.

Compressed air generation was a dominant contributor to environmental impacts in all scenarios, particularly under low-efficiency compressor conditions. Therefore, improving energy efficiency in air systems and optimizing foam separation can together yield significant sustainability gains.

In conclusion, while multistage FF systems provide strong environmental benefits, a well-optimized one-stage FF system with low foam fraction offers a promising and cost-effective alternative. System design and operational parameters should be co-optimized to balance performance, cost, and environmental impact in future large-scale PFAS treatment applications.

## Supplementary Material



## Notes

Environmental impact categories analyzed in this study; unit environmental impact of 1 m^3^ compressed air generated under various power and pressure conditions; capital construction costs to treat 1000 m^3^ PFAS-contaminated landfill leachate in the one-stage FF system from various system design sources; normalized environmental impact between one-stage FF system and three-stage FF system; normalized unit environmental impacts of compressed air under different generation conditions (compressed air generated under >30 kW and 6 bar gauge is as the reference); Appendix: input/output parameters and economic cost data obtained using the Work Breakdown Structure-Based Cost Model for Multi-Stage Bubble Aeration Drinking Water Treatment from the U.S. EPA Web site (PDF)
