# Supplementary material for: Life Cycle Assessment and Life Cycle Costing Analysis for Removing Per- and Polyfluoroalkyl Substances from Landfill Leachate with Foam Fractionation Technology
Source: ACS ES T Water. 2025 Oct 22;5(11):6289–98. doi: 10.1021/acsestwater.5c00381 (PMC12624725; doi:10.1021/acsestwater.5c00381)
Supplement: Supplementary file 1 [file ew5c00381_si_001.pdf]

Supporting information:

Life Cycle Assessment and Life Cycle Costing Analysis for Removing Per- and polyfluoroalkyl substances from Landfill Leachate with Foam Fractionation Technology

Gengyang Li [a], Yifei Wang [b], Qingguo Huang [b], Mason Peng [a], Ke Li [a]*

[a] College of Engineering, University of Georgia, Athens, GA 30602, United States

[b] College of Agricultural and Environmental Sciences, Griffin, GA 30223, United States

*Corresponding author: Ke Li

*E-mail: lukeli@uga.edu Telephone: 1-706-542-2201

6 Tables and 2 Figures

Table S1: Environmental impact categories analyzed in this study

Impact Category	Units
Ozone Depletion (OD)	kg CFC-11 eq
Global Warming Potential (GWP)	kg CO_2 eq.
Smog Formation (SF)	kg O_3 eq.
Acidification (AD)	mol H^+ eq.
Marine Eutrophication (ME)	kg N eq.
Non-Carcinogenics (NC)	CTUh
Respiratory Effects (RE)	kg PM_{10} eq
Ecotoxicity (ET)	CTUe

Table S2. Environmental impact results for treating 1000 m^3 PFASs-contaminated landfill leachate for one-stage FF system with 20% foam fraction

Impact category	Capital construction	Electricity for vacuum	Compressed air	Disposal	Total	Unit
OD	1.00E-05	5.51E-07	1.80E-05	2.74E-05	5.60E-05	kg CFC-11 eq
GWP	1.35E+01	1.21E+01	3.07E+02	4.85E+02	8.18E+02	kg CO_2 eq
SF	1.03E-01	5.69E-01	1.28E+01	7.98E+00	2.15E+01	kg O_3 eq
AD	1.23E+00	4.28E+00	7.43E+01	3.12E+01	1.11E+02	mol H^+ eq
ME	1.45E-03	4.65E-02	2.42E+00	1.37E+00	3.84E+00	kg N eq
C	2.87E-07	6.68E-07	3.24E-05	7.54E-05	1.09E-04	CTUh
NC	5.44E-06	1.51E-06	7.30E-05	4.45E-05	1.24E-04	CTUh
RE	3.11E-03	1.31E-02	3.47E-01	1.26E-01	4.89E-01	kg PM_{10} eq
ET	4.04E+01	1.12E+01	5.27E+02	8.00E+02	1.38E+03	CTUe

Table S3: Environmental impact results for treating 1000 m^3 PFASs-contaminated landfill leachate for one-stage FF system with 1% foam fraction.

Impact category	Capital construction	Electricity for vacuum	Compressed air	Disposal	Total	Unit
OD	1.00E-05	5.51E-07	1.80E-05	1.37E-06	3.00E-05	kg CFC-11 eq
GWP	1.35E+01	1.21E+01	3.07E+02	2.42E+01	3.57E+02	kg CO_2 eq.
SF	1.03E-01	5.69E-01	1.28E+01	3.99E-01	1.39E+01	kg O_3 eq
AD	1.23E+00	4.28E+00	7.43E+01	1.56E+00	8.14E+01	mol H^+ eq
ME	1.45E-03	4.65E-02	2.42E+00	6.83E-02	2.54E+00	kg N eq
C	2.87E-07	6.68E-07	3.24E-05	3.77E-06	3.71E-05	CTUh
NC	5.44E-06	1.51E-06	7.30E-05	2.23E-06	8.22E-05	CTUh
RE	3.11E-03	1.31E-02	3.47E-01	6.30E-03	3.70E-01	kg PM_{10} eq
ET	4.04E+01	1.12E+01	5.27E+02	4.00E+01	6.19E+02	CTUe

Table S4. Environmental impact results for treating 1000 m^3 PFASs contaminated landfill leachate with three-stage FF system.

Impact category	Capital construction	Electricity for vacuum	Compressed air	Disposal	Total	Unit
OD	1.33E-05	6.61E-07	2.16E-05	8.23E-11	3.56E-05	kg CFC-11 eq
GWP	1.79E+01	1.45E+01	3.69E+02	1.45E-03	4.02E+02	kg CO_2 eq.
SF	1.36E-01	6.83E-01	1.54E+01	2.39E-05	1.62E+01	kg O_3 eq
AD	1.62E+00	5.14E+00	8.92E+01	9.35E-05	9.60E+01	mol H^+ eq
ME	1.93E-03	5.58E-02	2.91E+00	4.10E-06	2.97E+00	kg N eq
C	3.81E-07	8.02E-07	3.89E-05	2.26E-10	4.01E-05	CTUh
NC	7.21E-06	1.82E-06	8.76E-05	1.34E-10	9.66E-05	CTUh
RE	4.12E-03	1.57E-02	4.17E-01	3.78E-07	4.37E-01	kg PM_{10} eq
ET	5.35E+01	1.34E+01	6.33E+02	2.40E-03	7.00E+02	CTUe

Table S5: The unit environmental impact of 1m^3 compressed air generated under various power and pressure conditions

Compressed air, optimized generation, at compressor/RER U						
Impact category	>30kW, 6 bar gauge	>30kW, 7 bar gauge	>30kW, 8 bar gauge	<30kW, 8 bar gauge	<30kW, 12 bar gauge	Units
OD	4.19E-09	4.50E-09	4.81E-09	1.26E-08	1.41E-08	kg CFC-11 eq
GWP	7.15E-02	7.69E-02	8.22E-02	1.74E-01	1.99E-01	kg CO_2 eq
SF	2.99E-03	3.21E-03	3.43E-03	9.03E-03	1.01E-02	kg O_3 eq
AF	1.73E-02	1.86E-02	1.98E-02	5.91E-02	6.51E-02	mol H^+ eq
ME	5.64E-04	6.05E-04	6.47E-04	2.27E-03	2.47E-03	kg N eq
C	7.54E-09	8.04E-09	8.54E-09	6.32E-08	6.55E-08	CTUh
NC	1.70E-08	1.81E-08	1.93E-08	2.17E-07	2.22E-07	CTUh
RE	8.08E-05	8.66E-05	9.24E-05	4.43E-04	4.70E-04	kg PM_{10} eq
ET	1.23E-01	1.31E-01	1.39E-01	1.23E+00	1.26E+00	CTUe

Table S6: Life cycling costing assessment results for treating 1000 m^3 PFASs contaminated landfill leachate with one-stage and three-stage FF system

Category	One-stage ($)	Three-stage ($)
Capital construction cost	37.31	49.20
Operation cost (electricity)	12.27	15.36
Maintenance cost (labor)	27.78	46.0
Total	77.36	110.6

Table S7: The capital construction costs to treat 1000m^3 PFAS-contaminated landfill leachate in the one-stage FF system from various system design sources

Design sources	Capital construction cost for one-stage FF system ($ in 2023)
EPA model, 2022	37.31
Donald R.Woods, 2003	18.31
Westech, 2018	21.90
Minnesota Pollution Control Agency, 2023	161.6

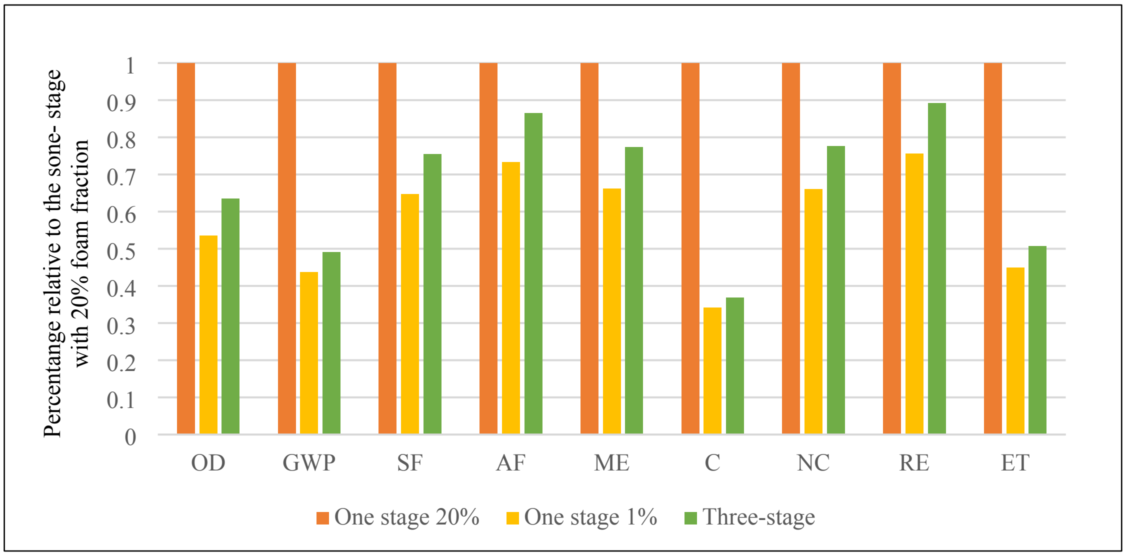

Figure S1: The life cycle environmental impact comparison for one-stage FF system (20% and 1%) and three-stage FF system. Impacts are normalized to one-stage FF system with 20% foam fraction.

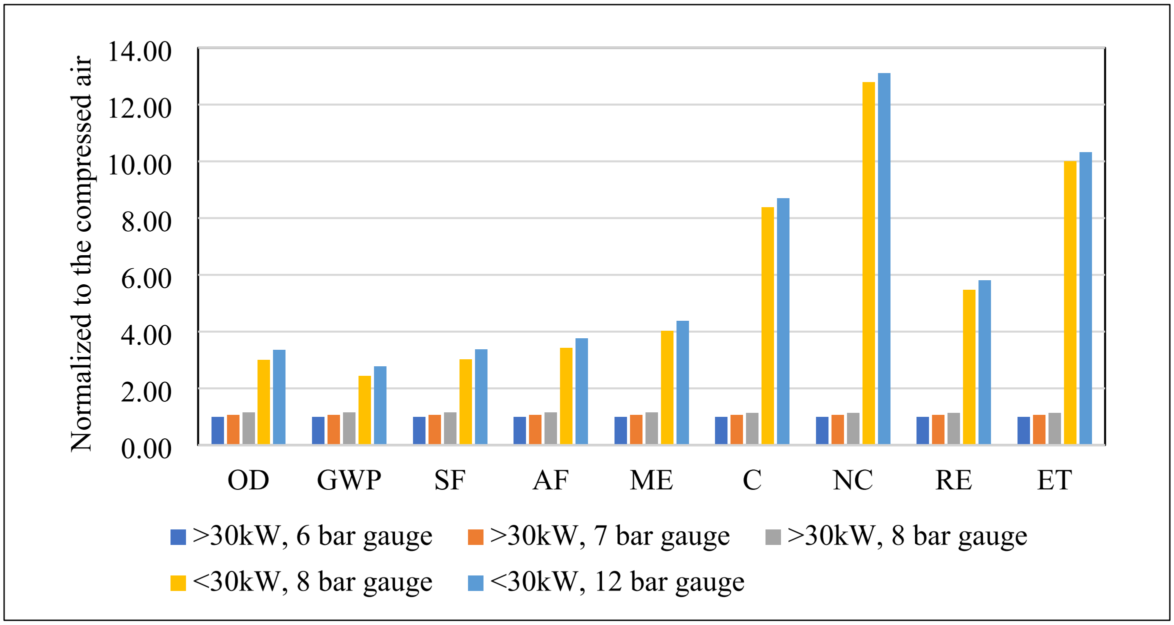

Figure S2. Normalized environmental impacts of 1m^3 compressed air under different generation conditions (compressed air generated under >30kW and 6 bar gauge is as the reference)

Appendix

Input/output parameters and economic cost data obtained using the Work Breakdown Structure-Based Cost Model for Multi-Stage Bubble Aeration Drinking Water Treatment from the U.S. EPA website.

Section 1 – Single stage FF system

Below include all input parameters for the Multi-Stage Bubble Aeration WBS model derived from the U.S. EPA. Spreadsheets used in this model are available under the "Drinking Water Treatment Technology Unit Cost Models" Drinking Water Treatment Technology Unit Cost Models | US EPA.

While this model was not used for every component of the life cycle cost assessment (e.g., electricity used for vacuum), sample outputs used in the costing model are included below for aeration basins, pumps, blowers, and piping (Figure 1.2), valves (Figure 1.3), instrumentation (Figure 1.4), building structures (Figure 1.5), and labor (Figure 1.6).

MULTI-STAGE BUBBLE AERATION SYSTEM DESIGN AND COST INPUT

Get Treatability Data

STEP 1

Select Contaminant — Other

For other contaminants, the buttons s[illegible]

STEP 2:

Select one of the eight standard designs at right OR select "CLEAR FOR MANUAL ENTRY"

- 0.030 MGD (21 gpm)
- 0.124 MGD (86 gpm)
- 0.305 MGD (212 gpm)
- 0.740 MGD (514 gpm)
- 2.152 MGD (1,494 gpm)
- 7.365 MGD (5,115 gpm)
- 22.614 MGD (15,704 gpm)
- 75.072 MGD (52,133 gpm)
- CLEAR FOR MANUAL ENTRY

Using a manual design

STEP 3:

(Optional for standard designs)

Enter or change values in the gold and blue cells below, under "Manual Inputs"

Input Complete -- Results Ready

STEP 4:

Results are ready (no need to click button)

Generate Results

MANUAL INPUTS

Cells in gold are required; cells in blue are optional

		Select units
**Design Flow**	**1.000**	MGD
**Average Flow**	**1**	MGD
**For information:**		
Treatment system design flow	**1.000**	MGD
Bypass design flow	**0.000**	MGD
		**Flow Input OK**

**Influent water concentration**	**29**	ug/L
**Contaminant removal input type**		<--- pick one
**Treated water concentration**	**0.07**	ug/L
Not required		*not required*
Not required		*not required*
Not required		*not required*
**Operating temperature**		
**Pilot rate constant**		1/min
**Pilot air intensity**		cfm/cubic ft
**For information:**		
Influent concentration	**29**	**ug/L**
Target treated water concentration		**ug/L**
Percent removal required		

Complete optional removal inputs above if you want to use the button below to optimize design

Design Type	pre-engineered package	<--- pick one
Number of stages	1	
Maximum water depth	10	feet
Optimize Design Air-to-water ratio	4.3	
Number of operating basins	16	units
For information:		
Theoretical percent removal achieved	NA	
Number of basins (including redundancy)	16	units
Basin length (including quiescent chamber)	9.5	feet
Basin width	3.5	feet
Basin height (including freeboard)	12	feet
Diffusers per stage	1	units
Total diffusers	16	units
		Basin Inputs OK
m3	9.50418	

VOC release at which air pollution control system is needed		lbs/day
For information:	Enter a release limit above to see guidance on off-gas control	
Off-gas pollution control technology	none	<--- pick one
Heat recovery type		*not required*
Spent GAC regeneration		*not required*
GAC bed life		*not required*
		Off-gas Inputs OK

Number of booster pumps		pumps
For information: # of booster pumps	1	pumps
Number of blowers		blowers
For information: # of blowers (including redundancy)	16	blowers
Number of redundant basins to be added		units
For information: Redundant basins	0	units
Component level		<-- pick or leave blank
System automation		<-- pick or leave blank
Include buildings?		<-- pick or leave blank
Include HVAC?		<-- pick or leave blank
Include land?		<-- pick or leave blank
For information: Component level	low cost	
Automation	manual	
		Optional Inputs OK

Figure 1.1. All input parameters used in the life cycle costing assessment model for the single stage FF system.

WBS #	Item	Design Quantity	Design Size	Size used in estimate	Unit Cost	Total Cost	Useful Life	Use?
**1.**	**Aeration Basins**							
1.1	Custom Designed							
1.1.1	*Concrete*	- units	NA	NA	NA	--	NA	
1.1.2	*Excavation*	- cy	NA	NA	NA	--	NA	
1.1.3	*Backfill and Compaction*	- cy	NA	NA	NA	--	NA	
1.1.4	*Steel Cover*	- sf	NA	NA	NA	--	NA	
1.1.5	*Membrane Baffles*	- units	NA	NA	NA	--	NA	
1.2	Prefabricated							
1.2.1	*Stainless Steel*	16 units	421 sf	421 sf	$ 62,155	$ 994,474	35	0
1.2.1	*Plastic with Stainless Steel Frame*	16 units	421 sf	421 sf	contact vendor	--	25	0
**1.2.1**	**Fiberglass**	**16 units**	**421 sf**	**421 sf**	**$ 38,226**	**$ 611,610**	**25**	**1**
1.3	Aerators							
**1.3.1**	**Diffusers**	**16 units**	**25 cfm**	**25 cfm**	**$ 79**	**$ 1,263**	**10**	**1**
**2.**	**Pumps and Blowers**							
**2.1**	**Booster Pumps**	**1 units**	**868 gpm**	**868 gpm**	**$ 22,347**	**$ 22,347**	**20**	**1**
2.2	Blowers							
2.2.1	*Centrifugal*	16 units	31 cfm	31 cfm	$ 14,110	$ 225,758	25	0
**2.2.1**	**Regenerative**	**16 units**	**31 cfm**	**31 cfm**	**$ 901**	**$ 14,423**	**25**	**1**
**3.**	**Piping**							
3.1	Process Piping							
3.1.1	*Ductile Iron*	100 lf	2.5 in. diam	2.5 in. diam	contact vendor	--	40	0
3.1.1	*CPVC*	100 lf	2.5 in. diam	2.5 in. diam	$ 13	$ 1,298	22	0
**3.1.1**	**PVC**	**100 lf**	**2.5 in. diam**	**2.5 in. diam**	**$ 4**	**$ 404**	**22**	**1**
3.1.1	*Stainless Steel*	100 lf	2.5 in. diam	2.5 in. diam	$ 119	$ 11,946	45	0
3.1.1	*Steel*	100 lf	2.5 in. diam	2.5 in. diam	$ 48	$ 4,842	35	0
3.2	Air Piping							
3.2.1	*Ductile Iron*	362 lf	2.5 in. diam	2.5 in. diam	contact vendor	--	40	0
3.2.1	*CPVC*	362 lf	2.5 in. diam	2.5 in. diam	$ 13	$ 4,699	22	0
**3.2.1**	**PVC**	**362 lf**	**2.5 in. diam**	**2.5 in. diam**	**$ 4**	**$ 1,461**	**22**	**1**
3.2.1	*Stainless Steel*	362 lf	2.5 in. diam	2.5 in. diam	$ 119	$ 43,245	45	0
3.2.1	*Steel*	362 lf	2.5 in. diam	2.5 in. diam	$ 48	$ 17,529	35	0
3.3	Influent and Treated Water Piping							
3.3.1	*Ductile Iron*	100 lf	6 in. diam	6 in. diam	$ 100	$ 9,979	40	0
3.3.1	*CPVC*	100 lf	6 in. diam	6 in. diam	$ 47	$ 4,661	22	0
**3.3.1**	**PVC**	**100 lf**	**6 in. diam**	**6 in. diam**	**$ 10**	**$ 973**	**22**	**1**
3.3.1	*Stainless Steel*	100 lf	6 in. diam	6 in. diam	$ 259	$ 25,856	45	0
3.3.1	*Steel*	100 lf	6 in. diam	6 in. diam	$ 109	$ 10,872	35	0

Figure 1.2 Output from the EPA WBS model using the above inputs for expenses of aeration basins, pumps, blowers, and piping.

**4.**	**Valves**							
4.2	Manual							
**4.2.1**	**Influent and treated water - Polypropylene/PVC**	**2 units**	**6 in. diam**	**6 in. diam**	**$ 820**	**$ 1,639**	**25**	**1**
4.2.1	*Influent and treated water - Stainless Steel*	2 units	6 in. diam	6 in. diam	$ 1,429	$ 2,857	25	0
4.2.1	*Influent and treated water - Cast Iron*	2 units	6 in. diam	6 in. diam	$ 1,446	$ 2,892	25	0
**4.2.2**	**Process - Polypropylene/PVC**	**50 units**	**2.5 in. diam**	**2.5 in. diam**	**$ 301**	**$ 15,069**	**25**	**1**
4.2.2	*Process - Stainless Steel*	50 units	2.5 in. diam	2.5 in. diam	$ 635	$ 31,763	25	0
4.2.2	*Process - Cast Iron*	50 units	2.5 in. diam	2.5 in. diam	$ 579	$ 28,925	25	0
**4.2.3**	**Air - Polypropylene/PVC**	**16 units**	**2.5 in. diam**	**2.5 in. diam**	**$ 301**	**$ 4,822**	**25**	**1**
4.2.3	*Air - Stainless Steel*	16 units	2.5 in. diam	2.5 in. diam	$ 635	$ 10,164	25	0
4.2.3	*Air - Cast Iron*	16 units	2.5 in. diam	2.5 in. diam	$ 579	$ 9,256	25	0
4.2.4	*Bypass - Polypropylene/PVC*	- units	NA	NA	NA	--	NA	
4.2.4	*Bypass - Stainless Steel*	- units	NA	NA	NA	--	NA	
4.2.4	*Bypass - Cast Iron*	- units	NA	NA	NA	--	NA	
4.3	Check Valves							
**4.3.1**	**Treated water - Polypropylene/PVC**	**1 units**	**6 in. diam**	**6 in. diam**	**$ 1,226**	**$ 1,226**	**25**	**1**
4.3.1	*Treated water - Stainless Steel*	1 units	6 in. diam	6 in. diam	$ 2,809	$ 2,809	25	0
4.3.1	*Treated water - Cast Iron*	1 units	6 in. diam	6 in. diam	$ 2,863	$ 2,863	25	0
**4.3.2**	**Process - Polypropylene/PVC**	**1 units**	**2.5 in. diam**	**2.5 in. diam**	**$ 327**	**$ 327**	**25**	**1**
4.3.2	*Process - Stainless Steel*	1 units	2.5 in. diam	2.5 in. diam	$ 854	$ 854	25	0
4.3.2	*Process - Cast Iron*	1 units	2.5 in. diam	2.5 in. diam	$ 836	$ 836	25	0

Figure 1.3 Output from the EPA WBS model using the above inputs for valves expenses.

**5.**	**Instrumentation**							
5.1	Flow Meters - Influent and Treated Water							
5.1.1	*Orifice Plate*	1 units	6 in. diam	6 in. diam	$ 3,418	$ 3,418	15	0
**5.1.1**	**Propeller**	**1 units**	**6 in. diam**	**6 in. diam**	**$ 4,479**	**$ 4,479**	**15**	**1**
5.1.1	*Venturi*	1 units	6 in. diam	6 in. diam	$ 3,069	$ 3,069	15	0
5.1.1	*Magnetic*	1 units	6 in. diam	6 in. diam	$ 6,186	$ 6,186	15	0
5.2	Flow Meters - Process							
5.2.1	*Orifice Plate*	16 units	2.5 in. diam	2.5 in. diam	$ 1,972	$ 31,550	15	0
**5.2.1**	**Propeller**	**16 units**	**2.5 in. diam**	**2.5 in. diam**	**$ 2,873**	**$ 45,968**	**15**	**1**
5.2.1	*Venturi*	16 units	2.5 in. diam	2.5 in. diam	$ 2,394	$ 38,299	15	0
5.2.1	*Magnetic*	16 units	2.5 in. diam	2.5 in. diam	$ 4,349	$ 69,581	15	0
5.3	Air Flow Meters							
**5.3.1**	**Rotameter**	**16 units**	**NA**	**NA**	**$ 2,900**	**$ 46,401**	**15**	**1**
**5.4**	**High/low alarms (for contact basins)**	**16 units**	**NA**	**NA**	**$ 600**	**$ 9,603**	**15**	**1**
**5.5**	**pH Meters**	**2 units**	**NA**	**NA**	**$ 3,047**	**$ 6,094**	**15**	**1**
**5.6**	**Pressure Gauges**	**32 units**	**NA**	**NA**	**$ 268**	**$ 8,563**	**15**	**1**

Figure 1.4 Output from the EPA WBS model using the above inputs for instrumentation expenses.

**8.**	**Building Structures and HVAC**							
8.1	Building 1							
8.1.1	*Small Low Cost Shed*	- units	2460 sf	2460 sf	NA	--	N/A	
**8.1.1**	**Low Quality**	**1 units**	**2460 sf**	**2460 sf**	**$ 77.85**	**$ 191,517**	**40**	**1**
8.1.1	*Medium Quality*	1 units	2460 sf	2460 sf	$ 93.40	$ 229,767	40	0
8.1.1	*High Quality*	1 units	2460 sf	2460 sf	$ 130.13	$ 320,116	40	0
8.2	Heating System							
8.2.1	*Electric resistance*	1 zones	50 MBH	50 MBH	$ 23,098	$ 23,098	25	0
8.2.1	*Natural gas non-condensing furnace*	1 zones	50 MBH	50 MBH	$ 16,597	$ 16,597	25	0
**8.2.1**	**Natural gas condensing furnace**	**1 zones**	**50 MBH**	**50 MBH**	**$ 16,597**	**$ 16,597**	**25**	**1**
8.2.1	*Standard efficiency oil furnace*	1 zones	50 MBH	50 MBH	$ 19,131	$ 19,131	25	0
8.2.1	*Mid-efficiency oil furnace*	1 zones	50 MBH	50 MBH	$ 19,131	$ 19,131	25	0

Figure 1.5 Output from the EPA WBS model using the above inputs for expenses of building structures.

Item	Quantity	Unit Cost	Total Cost ($/yr)	Use?
Labor				
Manager	264 hrs/yr	$ 57.93 /hr	$ 15,288	1
Administrative	264 hrs/yr	$ 32.90 /hr	$ 8,683	1
Operator	2,639 hrs/yr	$ 36.43 /hr	$ 96,145	1

Figure 1.6 Output from the EPA WBS model using the above inputs for labor expenses.

Section 2 – Three-stage FF system

Below include all input parameters for the Multi-Stage Bubble Aeration WBS model derived from the U.S. EPA. Spreadsheets used in this model are available under the "Drinking Water Treatment Technology Unit Cost Models" Drinking Water Treatment Technology Unit Cost Models | US EPA.

While this model was not used for every component of the life cycle cost assessment (e.g., electricity used for vacuum), sample outputs used in the costing model are included below for aeration basins, pumps, blowers, and piping (Figure 2.2), valves (Figure 2.3), instrumentation (Figure 2.4), building structures (Figure 2.5), and labor (Figure 2.6).

MULTI-STAGE BUBBLE AERATION SYSTEM DESIGN AND COST INPUT

Get Treatability Data

STEP 1
Select Contaminant

Other

For other contaminants, the buttons s

STEP 2:

Select one of the eight standard designs at right **OR** select "CLEAR FOR MANUAL ENTRY"

0.030 MGD (21 gpm)

0.124 MGD (86 gpm)

0.305 MGD (212 gpm)

0.740 MGD (514 gpm)

2.152 MGD (1,494 gpm)

7.365 MGD (5,115 gpm)

22.614 MGD (15,704 gpm)

75.072 MGD (52,133 gpm)

CLEAR FOR MANUAL ENTRY

Using a manual design

STEP 3:
(Optional for standard designs)
Enter or change values in the gold and blue cells below, under "Manual Inputs"

Input Complete -- Results Ready

STEP 4:

Results are ready (no need to click button)

Generate Results

MANUAL INPUTS
Cells in gold are required; cells in blue are optional

		Select units
**Design Flow**	1.000	MGD
**Average Flow**	1	MGD
For information:		
Treatment system design flow	1.000	MGD
Bypass design flow	0.000	MGD
		Flow Input OK

**Influent water concentration**	29	ug/L
**Contaminant removal input type**		<--- pick one
**Treated water concentration**	0.07	ug/L
Not required		*not required*
Not required		*not required*
Not required		*not required*
**Operating temperature**		
**Pilot rate constant**		1/min
**Pilot air intensity**		cfm/cubic ft
For information:		
Influent concentration	29	ug/L
Target treated water concentration		ug/L
Percent removal required		

Complete optional removal inputs above if you want to use the button below to optimize design

	Design Type	pre-engineered package	<--- pick one
	Number of stages	3	
	Maximum water depth	10	feet
Optimize Design	Air-to-water ratio	4.3	2039386.943
	Number of operating basins	28	units
	For information:		
	Theoretical percent removal achieved	NA	
	Number of basins (including redundancy)	28	units
	Basin length (including quiescent chamber)	6.5	feet
	Basin width	2.5	feet
	Basin height (including freeboard)	12	feet
	Diffusers per stage	1	units
	Total diffusers	84	units
			Basin Inputs OK
	m3	5.5224	

VOC release at which air pollution control system is needed		lbs/day
For information:	Enter a release limit above to see guidance on off-gas control	
Off-gas pollution control technology	none	<--- pick one
Heat recovery type		*not required*
Spent GAC regeneration		*not required*
GAC bed life		*not required*
		Off-gas Inputs OK

Number of booster pumps		pumps
For information: # of booster pumps	1	pumps
Number of blowers		blowers
For information: # of blowers (including redundancy)	28	blowers
Number of redundant basins to be added		units
For information: Redundant basins	0	units
Component level		<-- pick or leave blank
System automation		<-- pick or leave blank
Include buildings?		<-- pick or leave blank
Include HVAC?		<-- pick or leave blank
Include land?		<-- pick or leave blank
For information: Component level	low cost	
Automation	manual	
		Optional Inputs OK

Figure 2.1. All input parameters used in the life cycle costing assessment model for the three-stage FF system.

WBS #	Item	Design Quantity	Design Size	Size used in estimate	Unit Cost	Total Cost	Useful Life	Use?
1.	**Aeration Basins**							
1.2	Prefabricated							
1.2.1	*Stainless Steel*	28 units	339 sf	339 sf	$ 54,101	$ 1,514,822	35	0
1.2.1	*Plastic with Stainless Steel Frame*	28 units	339 sf	339 sf	contact vendor	--	25	0
**1.2.1**	**Fiberglass**	**28 units**	**339 sf**	**339 sf**	**$ 31,544**	**$ 883,242**	**25**	**1**
1.3	Aerators							
**1.3.1**	**Diffusers**	**84 units**	**5 cfm**	**5 cfm**	**$ 46**	**$ 3,898**	**10**	**1**
2.	**Pumps and Blowers**							
**2.1**	**Booster Pumps**	**1 units**	**868 gpm**	**868 gpm**	**$ 22,347**	**$ 22,347**	**20**	**1**
2.2	Blowers							
2.2.1	*Centrifugal*	28 units	18 cfm	18 cfm	$ 13,817	$ 386,874	25	0
**2.2.1**	**Regenerative**	**28 units**	**18 cfm**	**18 cfm**	**$ 629**	**$ 17,620**	**25**	**1**
3.	**Piping**							
3.1	Process Piping							
3.1.1	*Ductile Iron*	100 lf	2 in. diam	2 in. diam	contact vendor	--	40	0
3.1.1	*CPVC*	100 lf	2 in. diam	2 in. diam	$ 10	$ 980	22	0
**3.1.1**	**PVC**	**100 lf**	**2 in. diam**	**2 in. diam**	**$ 3**	**$ 342**	**22**	**1**
3.1.1	*Stainless Steel*	100 lf	2 in. diam	2 in. diam	$ 95	$ 9,544	45	0
3.1.1	*Steel*	100 lf	2 in. diam	2 in. diam	$ 39	$ 3,940	35	0
3.2	Air Piping							
3.2.1	*Ductile Iron*	1,072 lf	2 in. diam	2 in. diam	contact vendor	--	40	0
3.2.1	*CPVC*	1,072 lf	2 in. diam	2 in. diam	$ 10	$ 10,507	22	0
**3.2.1**	**PVC**	**1,072 lf**	**2 in. diam**	**2 in. diam**	**$ 3**	**$ 3,664**	**22**	**1**
3.2.1	*Stainless Steel*	1,072 lf	2 in. diam	2 in. diam	$ 95	$ 102,313	45	0
3.2.1	*Steel*	1,072 lf	2 in. diam	2 in. diam	$ 39	$ 42,239	35	0
3.3	Influent and Treated Water Piping							
3.3.1	*Ductile Iron*	100 lf	6 in. diam	6 in. diam	$ 100	$ 9,979	40	0
3.3.1	*CPVC*	100 lf	6 in. diam	6 in. diam	$ 47	$ 4,661	22	0
**3.3.1**	**PVC**	**100 lf**	**6 in. diam**	**6 in. diam**	**$ 10**	**$ 973**	**22**	**1**
3.3.1	*Stainless Steel*	100 lf	6 in. diam	6 in. diam	$ 259	$ 25,856	45	0
3.3.1	*Steel*	100 lf	6 in. diam	6 in. diam	$ 109	$ 10,872	35	0

Figure 2.2 Output from the EPA WBS model using the above inputs for expenses of aeration basins, pumps, blowers, and piping.

4.	**Valves**							
4.2	Manual							
**4.2.1**	**Influent and treated water - Polypropylene/PVC**	**2 units**	**6 in. diam**	**6 in. diam**	**$ 820**	**$ 1,639**	**25**	**1**
4.2.1	*Influent and treated water - Stainless Steel*	2 units	6 in. diam	6 in. diam	$ 1,429	$ 2,857	25	0
4.2.1	*Influent and treated water - Cast Iron*	2 units	6 in. diam	6 in. diam	$ 1,446	$ 2,892	25	0
**4.2.2**	**Process - Polypropylene/PVC**	**86 units**	**2 in. diam**	**2 in. diam**	**$ 252**	**$ 21,636**	**25**	**1**
4.2.2	*Process - Stainless Steel*	86 units	2 in. diam	2 in. diam	$ 517	$ 44,435	25	0
4.2.2	*Process - Cast Iron*	86 units	2 in. diam	2 in. diam	$ 458	$ 39,392	25	0
**4.2.3**	**Air - Polypropylene/PVC**	**28 units**	**2 in. diam**	**2 in. diam**	**$ 252**	**$ 7,044**	**25**	**1**
4.2.3	*Air - Stainless Steel*	28 units	2 in. diam	2 in. diam	$ 517	$ 14,467	25	0
4.2.3	*Air - Cast Iron*	28 units	2 in. diam	2 in. diam	$ 458	$ 12,825	25	0
4.3	Check Valves							
**4.3.1**	**Treated water - Polypropylene/PVC**	**1 units**	**6 in. diam**	**6 in. diam**	**$ 1,226**	**$ 1,226**	**25**	**1**
4.3.1	*Treated water - Stainless Steel*	1 units	6 in. diam	6 in. diam	$ 2,809	$ 2,809	25	0
4.3.1	*Treated water - Cast Iron*	1 units	6 in. diam	6 in. diam	$ 2,863	$ 2,863	25	0
**4.3.2**	**Process - Polypropylene/PVC**	**1 units**	**2 in. diam**	**2 in. diam**	**$ 247**	**$ 247**	**25**	**1**
4.3.2	*Process - Stainless Steel*	1 units	2 in. diam	2 in. diam	$ 663	$ 663	25	0
4.3.2	*Process - Cast Iron*	1 units	2 in. diam	2 in. diam	$ 715	$ 715	25	0

Figure 2.3 Output from the EPA WBS model using the above inputs for valves expenses.

5.	**Instrumentation**							
5.1	Flow Meters - Influent and Treated Water							
5.1.1	*Orifice Plate*	1 units	6 in. diam	6 in. diam	$ 3,418	$ 3,418	15	0
**5.1.1**	**Propeller**	**1 units**	**6 in. diam**	**6 in. diam**	**$ 4,479**	**$ 4,479**	**15**	**1**
5.1.1	*Venturi*	1 units	6 in. diam	6 in. diam	$ 3,069	$ 3,069	15	0
5.1.1	*Magnetic*	1 units	6 in. diam	6 in. diam	$ 6,186	$ 6,186	15	0
5.2	Flow Meters - Process							
5.2.1	*Orifice Plate*	28 units	2 in. diam	2 in. diam	$ 1,731	$ 48,473	15	0
**5.2.1**	**Propeller**	**28 units**	**2 in. diam**	**2 in. diam**	**$ 2,615**	**$ 73,234**	**15**	**1**
5.2.1	*Venturi*	28 units	2 in. diam	2 in. diam	$ 2,310	$ 64,682	15	0
5.2.1	*Magnetic*	28 units	2 in. diam	2 in. diam	$ 4,096	$ 114,678	15	0
5.3	Air Flow Meters							
**5.3.1**	**Rotameter**	**28 units**	**NA**	**NA**	**$ 2,900**	**$ 81,202**	**15**	**1**
**5.4**	**High/low alarms (for contact basins)**	**28 units**	**NA**	**NA**	**$ 600**	**$ 16,805**	**15**	**1**
**5.5**	**pH Meters**	**2 units**	**NA**	**NA**	**$ 3,047**	**$ 6,094**	**15**	**1**
**5.6**	**Pressure Gauges**	**56 units**	**NA**	**NA**	**$ 268**	**$ 14,984**	**15**	**1**
5.7	*Electrical Enclosure*	- units	NA	NA	NA	--	NA	

Figure 2.4 Output from the EPA WBS model using the above inputs for instrumentation expenses.

8.	**Building Structures and HVAC**							
8.1	Building 1							
8.1.1	*Small Low Cost Shed*	- units	2330 sf	2330 sf	NA	--	N/A	
**8.1.1**	**Low Quality**	**1 units**	**2330 sf**	**2330 sf**	**$ 78.26**	**$ 182,336**	**40**	**1**
8.1.1	*Medium Quality*	1 units	2330 sf	2330 sf	$ 93.89	$ 218,765	40	0
8.1.1	*High Quality*	1 units	2330 sf	2330 sf	$ 131.10	$ 305,458	40	0
8.2	Heating System							
8.2.1	*Electric resistance*	1 zones	46 MBH	46 MBH	$ 21,630	$ 21,630	25	0
8.2.1	*Natural gas non-condensing furnace*	1 zones	46 MBH	46 MBH	$ 15,567	$ 15,567	25	0
**8.2.1**	**Natural gas condensing furnace**	**1 zones**	**46 MBH**	**46 MBH**	**$ 15,567**	**$ 15,567**	**25**	**1**
8.2.1	*Standard efficiency oil furnace*	1 zones	46 MBH	46 MBH	$ 17,994	$ 17,994	25	0
8.2.1	*Mid-efficiency oil furnace*	1 zones	46 MBH	46 MBH	$ 17,994	$ 17,994	25	0

Figure 2.5 Output from the EPA WBS model using the above inputs for expenses of building structures.

Item	Quantity	Unit Cost	Total Cost ($/yr)	Use?
Labor				
Manager	437 hrs/yr	$ 57.93 /hr	$ 25,334	1
Administrative	437 hrs/yr	$ 32.90 /hr	$ 14,388	1
Operator	4,373 hrs/yr	$ 36.43 /hr	$ 159,320	1

Figure 2.6 Output from the EPA WBS model using the above inputs for labor expenses.
